# Glycan–Lectin Interactions in Cancer and Viral Infections and How to Disrupt Them

**DOI:** 10.3390/ijms221910577

**Published:** 2021-09-30

**Authors:** Stefanie Maria Kremsreiter, Ann-Sophie Helene Kroell, Katharina Weinberger, Heike Boehm

**Affiliations:** 1 Institute for Pharmacy and Molecular Biotechnology (IPMB), Ruprecht Karls University Heidelberg, Im Neuenheimer Feld 364, 69120 Heidelberg, Germany; Stefanie.Kremsreiter@stud.uni-heidelberg.de (S.M.K.); ann-sophie@kroell.de (A.-S.H.K.); weinberger@stud.uni-heidelberg.de (K.W.); 2 Max-Planck-Institute for Medical Research, Jahnstr. 29, 69120 Heidelberg, Germany

**Keywords:** cancer, DC-SIGN, galectins, glycosylation, lectins, MGL, selectins, virus

## Abstract

Glycan–lectin interactions play an essential role in different cellular processes. One of their main functions is involvement in the immune response to pathogens or inflammation. However, cancer cells and viruses have adapted to avail themselves of these interactions. By displaying specific glycosylation structures, they are able to bind to lectins, thus promoting pathogenesis. While glycan–lectin interactions promote tumor progression, metastasis, and/or chemoresistance in cancer, in viral infections they are important for viral entry, release, and/or immune escape. For several years now, a growing number of investigations have been devoted to clarifying the role of glycan–lectin interactions in cancer and viral infections. Various overviews have already summarized and highlighted their findings. In this review, we consider the interactions of the lectins MGL, DC-SIGN, selectins, and galectins in both cancer and viral infections together. A possible transfer of ways to target and disrupt them might lead to new therapeutic approaches in different pathological backgrounds.

## 1. Introduction

Glycosylation is a multistep process involving the covalent addition of saccharides to proteins or lipids at the endoplasmic reticulum and Golgi. It is one of the most common and diverse forms of the post-translational modification of proteins [1,2]. The main types include N-linked glycosylation (glycans are attached to nitrogen of asparagine) and O-linked glycosylation (glycans are attached to the hydroxyl group of serine or threonine). Moreover, the formation of oligo- and polysaccharides is defined by the monomer sequence as well as the linkage (which can be α or β), whereas the position of the linkage is highly variable [3,4]. Glycosylation has a major impact on structural and modulatory functions as well as cellular interactions [2].

For cell–cell or cell–pathogen interactions, glycans bind to glycan-binding proteins named lectins. Lectins are a large protein class which display a characteristic carbohydrate recognition domain (CRD) in order to bind specific glycosylation structures [2]. Glycan–lectin interactions mediate many different biological processes, including cell–cell recognition, developmental processes, and protection against pathogens [1]. Even small changes in carbohydrate moieties may strongly affect interaction and consequent functionality [5]. In this review, we will focus on the interactions of glycans with four different lectins, namely galectins and three different C-type lectin receptors (CLRs) including the macrophage galactose binding lectin (MGL), the dendritic cell-specific intracellular adhesion molecule 3-grabbing nonintegrin (DC-SIGN), and selectins. We selected these four lectins because it has been shown that all four receptors play a key role in cancer [6,7] and in viral infection [8,9] by interacting with disease-specific glycosylation patterns.

**Figure 1:**
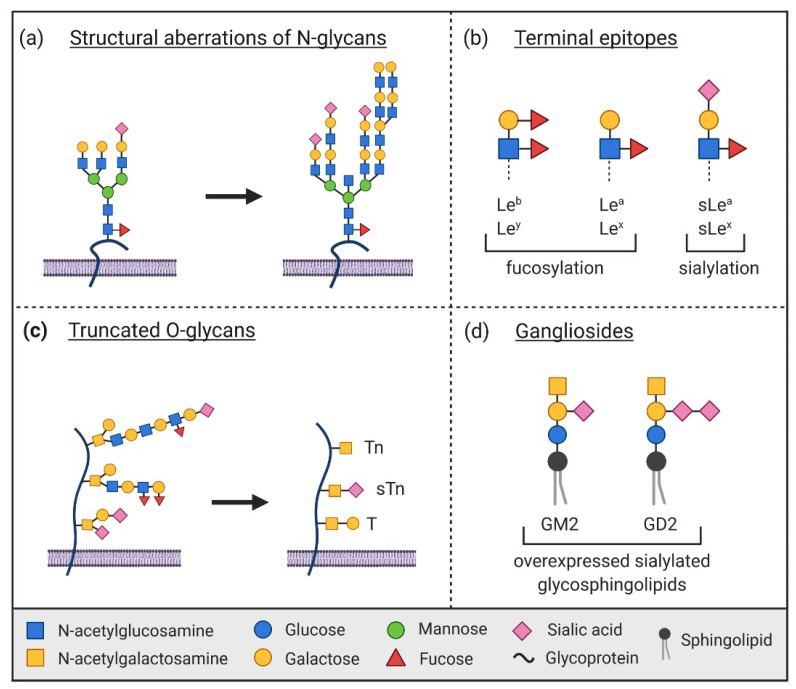
Common aberrations of glycan structures in cancer. (**a**) Altered and increased branching of N-glycans. (**b**) Capping of glycan structures with terminal epitopes such as the fucosylated Lewis (Le) antigen or sialylated structures like the sialyl Lewis (sLe) antigen. (**c**) Expression of truncated O-glycans like the monosaccharide GalNAc (Tn antigen), the sialylated Tn antigen (sTn), or the Thomsen Friedenreich antigen (T antigen). (**d**) Overexpression of gangliosides, a family of sialylated glycosphingolipids—for example, monosialoganglioside GM2 and disialoganglioside GD2. The figure was created with BioRender.com and is adapted from Mereiter et al., 2019 [10].

Aberrant glycosylation is a characteristic feature of cancer cells, as it is involved in many cancer-related pathways [10,11]. Incomplete synthesis of glycan structures and the formation of new structures promote a tumor-specific glyco-code [12]. This glyco-code includes different highly branched N-glycans and truncated O-glycans as well as various terminal fucosylated or sialylated glycans or glycosphingolipids [10,12,13] (Figure 1). Increased α2,6-sialylation is a critical aberration observed in cancer development that contributes to increasing the metastatic growth potential of tumor cells. Due to the altered ratio between α2,3- and α2,6-linked sialic acids, several of those glycans were found to be relevant for diagnosis [14]. By binding to lectins, the tumor-specific glycans influence key biological processes including, for example, cell adhesion, signaling, and proliferation. These processes can be connected to several essential cancer hallmarks like activating invasion and metastasis, proliferative signaling, and/or avoiding immune destruction [7,10,12].

In viruses, especially enveloped viruses, glycans play also an important role by, for example, enhancing viral infectivity [15]. Since viruses are deficient in proteins such as glycosyltransferases, they exploit the cellular glycosylation machinery [16]. However, instead of undergoing the conventional process of host cell glycoproteins (GPs), viral proteins might break out of the machinery early or translocate, resulting in the formation of virus-specific glycans [17]. By interacting with lectins, these viral GPs can promote virulence, for example, by enabling viral attachment and host cell uptake, viral release, or immune evasion [8,17].

This review covering a range of four different lectins (MGL, DC-SIGN, selectins, and galectins) provides a basic overview of current knowledge for those who are new to the glycan research field or interested in looking at glycan–lectin interactions in cancer and viral infections. Some promising therapeutic approaches are based on disrupting these interactions. For each receptor, we first present the physiological role of the receptor by addressing possible classifications, their structure, ligands, expression patterns, and their function. It is then shown how the expression of the receptor or glycan is altered in cancer. We present the tumor-promoting effects of these interactions as well as preclinically or clinically investigated strategies targeting these interactions. Similarly, we describe the role of lectins interacting with glycan patterns or GPs of well-known viruses including human immunodeficiency virus (HIV), Ebola virus (EBOV), influenza virus, and the severe acute respiratory syndrome coronavirus 2 (SARS-CoV-2). We show their role in promoting infection and discuss potential therapeutic strategies. By comparing glycan–lectin interactions in cancer and viral infections, we hope to find commonalities with regard to therapeutic approaches or potential clinical methods. Such commonalities could be the basis for further investigations with the goal of translating promising therapeutic approaches in either cancer or viral infections to the other disease.

## 2. Macrophage Galactose-Binding Lectin

**Figure 2:**
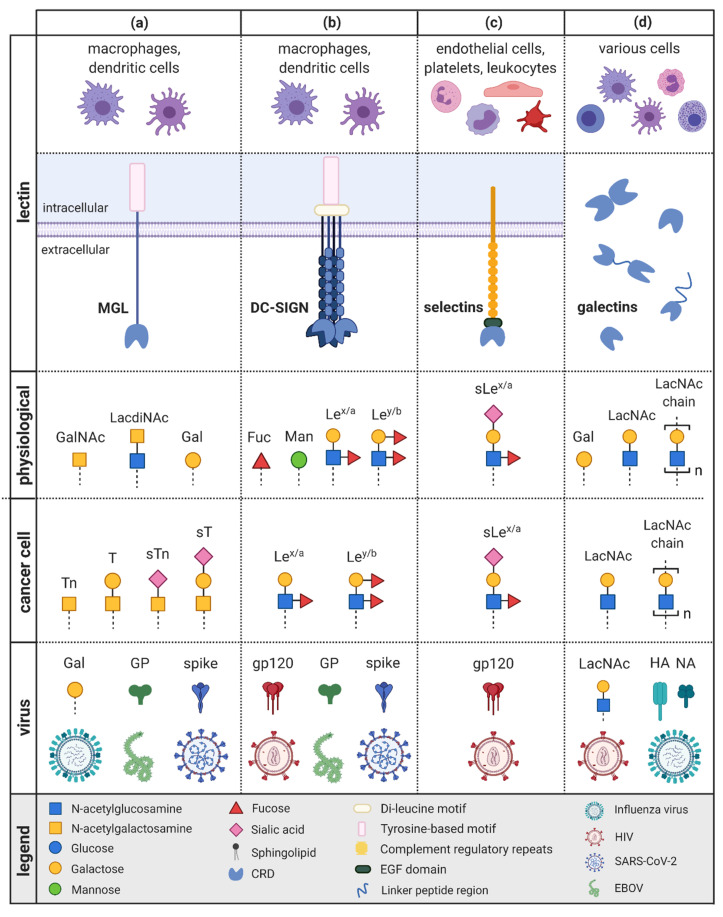
Lectins and their glycan interaction partner physiologically, in cancer and viral infections. The lectin subtypes MGL (**a**), DC-SIGN (**b**), and selectins (**c**) belong to the C-type lectin receptors (CLRs) and consist of a carbohydrate recognition domain (CRD) at the end of their extracellular domain as well as a transmembrane and a cytoplasmatic tail. Galectins (**d**) structurally possess one or two CRDs and, in part, a non-CRD domain or a linker peptide region. (**a**) MGL is expressed on macrophages and dendritic cells and physiologically binds galactose-terminated glycans and *N*-acetyl-galactosamine (GalNAc). In cancer, MGL interacts with truncated O-glycans such as the T and Tn antigen as well as their sialylated forms sT and sTn. During viral infection, MGL binds terminal galactose-containing glycans of influenza virus, the viral envelope glycoprotein (GP) of Ebola virus (EBOV), and the highly glycosylated spike protein of SARS-CoV-2. (**b**) DC-SIGN is also expressed on macrophages and DCs and physiologically recognizes mannose- and fucose-containing glycans including the blood-group Lewis antigen family (Le) on pathogens. On cancer cells, DC-SIGN interacts, thus, mainly with the highly expressed Lewis glyco-epitopes. Furthermore, this lectin detects a wide range of viral glycoproteins, including high-mannose residues on the gp120 of HIV, high-mannose glycans of EBOV, as well as high-mannose and Le epitopes on the spike protein of SARS-CoV-2. (**c**) Selectins are found on endothelial cells, platelets, and leukocytes and recognize sialyl Le antigen x (sLe^x^) and its isomer sialyl Le antigen a (sLe^a^). The major ligands of selectins on cancer cells are thus the overexpressed sLe^x/a^. In the context of viral infections, the glycoprotein gp120 in HIV was found to play a key role in the interaction with L selectin on T cells. (**d**) Galectins are expressed by various cells of the innate as well as the adaptive immune system and physiologically bind β-galactose-containing glycoconjugates. In this context, *N*-acetyllactosamine (LacNAc) represents the minimal binding motif. Cancer cells possess a high number of terminal LacNAc as well as LacNAc chains resulting in increased affinity for most galectins. Enhanced LacNAc residues are also present in HIV infections. In addition, galectin-1 interacts with hemagglutinin (HA) and, possibly, neuraminidase (NA), two surface glycoproteins of the influenza virus. The figure was created with BioRender.com.

MGL belongs to a subgroup of type II CLRs, the galactose-type lectins [18]. CLRs have the property to bind carbohydrates in a calcium-dependent manner [19] and are divided into 14 different types based on their domain structures [20,21]. MGL consists of a single C-type, lectin-like domain (CTLD), an extracellular stalk region, a transmembrane region, and, finally, a cytoplasmic tail containing a tyrosine-based signaling motif which regulates endocytosis activity. The CRD is located inside the CTLD [22] (Figure 2a). This galactose-type lectin binds specifically to galactose-terminated glycans, in particular, terminal α- and β-linked *N*-acetylgalactosamine (GalNAc) residues [23]. This binding is due to a specific amino acid sequence, which in this case is glutamine-proline-aspartic acid (QPD) [18]. Another ligand is LacdiNAc, which consists of GalNAc linked to *N*-acetylglucosamine (GlcNAc) via β 1-4 linkage (GalNAcβ1-4GlcNAc) [18].

MGL is mainly expressed by macrophages as well as immature dendritic cells (iDCs) [7,18]. With the help of MGL, these antigen-presenting cells (APCs) are able to recognize pathogens and internalize glycosylated antigens in order to present them on their surface through the major histocompatibility complex classes I and II (MHCI + II) [18]. Thus, it is involved in the innate and adaptive immune response [23]. Furthermore, this receptor also plays a role in the regulation of T cell homeostasis [22,24]. MGL is a multifaceted receptor that can bind more than one ligand, thereby triggering different types of immune responses [22].

### 2.1. MGL in Cancer

The main ligands of MGL in cancer are truncated O-glycans including the Tn antigen (α-GalNAc-Ser/Thr) [18,25] and the T antigen (Galβ1–3GalNAc) [26] as well as their sialylated versions [22]. The tumor antigen mucin 1 (MUC1) expresses these shortened sugar structures on its surface [27]. Moreover, MUC1 is overexpressed in many cancer types, including colorectal, cervical, and breast cancer cells [28,29], and is hypoglycosylated compared to normal epithelia [30]. The binding of MGL to the truncated glycan structures on MUC1 leads to a defective T-helper (Th) cell-mediated response as well as to a reduction of cytotoxic T lymphocytes (CTLs) [22], which are usually able to recognize foreign antigens presented via MHCI and eliminate cancer cells [31]. In addition, the tumor-associated MUC1 glycoform bearing the sialyl T antigen was shown to impair the differentiation of DCs and impede their ability to produce interleukin-12 (IL-12) [32]. IL-12 is an inflammatory cytokine that plays an important role in innate and adaptive immunity by enhancing natural killer (NK) cell cytotoxicity and inducing the production of interferon-γ by T cells and NK cells [33,34]. Besides a reduction of IL-12, anti-inflammatory IL-10 production by DCs is increased in the cancer microenvironment [7], leading to impaired DC function by enhancing spontaneous apoptosis or defective antigen presentation [32]. As a result, the binding of MGL to altered glycosylation prevents an antitumor immune response, allowing cancer cells to escape the immune system.

Some therapeutic approaches that aim to prevent the described immune escape by targeting glycan–lectin interaction are being investigated. In preclinical studies, Tn-MUC1 chimeric antigen receptor (CAR) T cells could control tumor growth in pancreatic cancer as well as in leukemia xenograft models without showing reactivity against normal tissue cells [35]. Other studies revealed the promising use of cancer immunotherapy involving a monoclonal antibody (5E5) that targets the aberrant Tn glycoform of MUC1 and is able to kill the tumor cell [36]. Napoletano et al. (2012) showed that an anti-MGL antibody leads to a CD8+ T cell immune response by activating different signal pathways including ERK1,2 and NF-κB. Consequently, DCs are able to mature and trigger T cell activation [37]. The other ligand, LacdiNAc, can be used as a glyco-biomarker for, for example, colon, ovarian, and lung cancer, as elevated LacdiNAc levels have been observed on glycoproteins during tumor development [38].

### 2.2. MGL in Viral Infections

Apart from its known functions in tumor progression, MGL was found to also play an important role in the context of viral infections. Influenza virus glycans carry terminal galactose [39], and thus represent potential ligands for galactose-binding lectins such as MGL. It was observed in mice that MGL acts as an attachment and infectious entry receptor for influenza viruses into macrophages [40,41].

Furthermore, MGL interacts with the viral envelope GP of Ebola virus (EBOV), which contains an O glycan-rich, mucin-like domain [22,42,43]. The expression of MGL causes enhanced virus infectivity by improving viral attachment to the native cellular receptors [42]. Since MGL prefers binding to O-glycans by adding extended N-glycans, the interaction between GPs and MGL is disrupted and infectivity could be reduced [44]. Moreover, several studies showed that GP-specific neutralizing antibodies successfully protect animals from EBOV infection [45,46,47,48,49], which demonstrates that the viral envelope GP is a useful therapeutic target for EBOV infection. Currently, there are two approved vaccines against EBOV containing the gene of the Ebola virus that encodes for the surface GP. The first one is a recombinant vesicular stomatitis virus-based vaccine (VSV-EBOV, also known as Ervebo) ([50,51], reviewed in [52]); the second is the combination vaccine Ad26.ZEBOV/MVA BN Filo, available since 2020 [53].

Additionally, MGL might be a potential entry receptor for SARS-CoV-2, as it was shown that its spike protein is highly glycosylated [54] and that the glycans could serve as MGL ligands [55]. Thus, disrupting the interaction with MGL could be a beneficial therapeutic target (described in detail in [56]).

## 3. Dendritic Cell-Specific ICAM3-Grabbing Non-Integrin

DC-SIGN belongs to the mannose type of CLR and, like MGL, is part of the type II group of CLRs. Thus, it also comprises a stalk region located extracellularly, a transmembrane domain, as well as a cytoplasmic tail [22,57,58]. This cytoplasmic region contains a tyrosine-based and a di-leucine motif that are important for antigen uptake [59] (Figure 2b). In addition, the CRD comprises the amino acid sequences of glutamic acid, proline, and asparagine (EPN) [60], which is responsible for the recognition of high-mannose [61] and fucose-containing glycans, including the blood-group Lewis antigen family [62,63]. DC-SIGN is mainly expressed by iDC and macrophages [64,65]. One of the main tasks of DC-SIGN is the detection, internalization, and presentation of pathogens through DCs [66,67]. Furthermore, DC-SIGN enables the interactions of DCs and T cells by binding to the T cell surface receptor ICAM3 [65].

### 3.1. DC-SIGN in Tumor Escape and Progression

DC-SIGN is known to bind tumor-associated glycans, the so-called Lewis antigens (Le^x^, Le^y^, Le^a^, Le^b^), which are highly upregulated in cancer [68]. They consist of fucose units bound to a disaccharide of GlcNAc and galactose [7].

In colon cancer, for example, the tumor-associated carcinoembryonic antigen (CEA) is overexpressed and carries Lewis antigens. DCs bind to the overexpressed Lewis antigens on the CEAs of colorectal cancer cells but not to the CEAs of non-tumor cells. To evade an immune response, tumor cells inhibit the original functions of DCs. By interacting with DC-SIGN, Lewis antigens increase the production of IL-10 [64,69], an anti-inflammatory cytokine, which has the effect of preventing DCs from maturing. Consequently, iDCs are unable to contribute to a strong T cell response and T cell tolerance is induced instead [70], as observed in primary cutaneous T cell lymphoma [71]. Thus, cancer cells are able to suppress the immune response [7]. 

DC-SIGN is not only involved in tumor immune evasion, but also promotes tumor development. In follicular lymphoma, DC-SIGN activates the IgM follicular lymphoma B cell receptor by binding to this highly mannosylated receptor, thereby leading to its activation [72]. This supports the growth of malignant B cells [73]. Moreover, DC-SIGN on macrophages interacts with semen clusterin, an overexpressed GP in human luminal breast cancer, which expresses the terminal fucosylated glycans (Le^x^ and Le^y^) on its surface [74], thereby promoting carcinogenesis and tumor progression [75,76]. On the one hand, this interaction restricts the antigen presentation of macrophages, while, on the other, it stimulates the differentiation of macrophages into a pro-angiogenic type [75].

DC-SIGN serves as a promising target molecule for DC-based vaccination strategies, as demonstrated in several studies [77,78,79]. Targeting DCs by glycan-modified antigens or antibodies in combination with the transient deletion of Treg cells has resulted in improved antigen-specific CD4+ and CD8+ T cell responses and long-term tumor regression [80]. By using an anti-DC-SIGN antibody (hD1V1G2/G4 or AZN-D1), an antigen-specific T cell response was observed [78,79]. Furthermore, when DC-SIGN is blocked by a neutralizing antibody on tumor-associated macrophages in bladder cancer, these cells no longer secrete anti-inflammatory cytokines. Instead, there is increased cytotoxic activity of T cells against tumor cells [81]. 

In addition, glycan targeting of the humanized anti Le^y^ mAb hu3S193 showed great potential due to its high specificity against Le^y^ as well as low toxicity but lacked the significant clinical efficacy needed to justify proceeding with phase III studies. Conjugating hu3S193 could considerably enhance efficacy but would still need further optimization [82,83,84,85].

### 3.2. DC-SIGN in Viral Infections

It is known that DC-SIGN detects a wide range of viruses, including HIV [86], filoviruses [87], SARS-CoV [88], and many more [8]. However, some viruses have adapted to use DCs to evade the immune response and infect other cells. HIV, for example, attaches to DC-SIGN via high-mannose residues on gp120 glycans and uses DCs as shuttles, allowing the trans-infection of neighboring CD4+ T cells [57,86]. Furthermore, DCs sequester HIV virions in multivesicular bodies, enabling the virus to evade immune response [89]. The binding of HIV to DC-SIGN enhances IL-10 (anti-inflammatory cytokine) production via the ERK signaling pathway [62,90]. To prevent IL-10 induction and the impairment of DC maturation, the binding of gp120 to DC-SIGN can be inhibited by using, for example, the neutralizing human monoclonal antibody 2G12 [90,91]. In addition, it has been shown that competitive inhibitors, such as multivalent dendrimeric Lewis-type antigen-based complexes or other synthetic carbohydrate constructs, can block the binding of gp120 to DC-SIGN, whereby HIV transmission to CD4+ T cells is prevented [92,93,94,95]. Moreover, the glycomimetic DC-SIGN ligand Polyman26 can inhibit DC-SIGN-mediated HIV infection [96]. Chloroquine [97] as well as dextran and gp120 antagonists [98] show a similar effect. siRNAs directed against DC-SIGN may also prevent the initial binding of HIV-1 to the receptor [99]. Moreover, it was demonstrated that semen clusterin is not only a promising ligand for DC-SIGN in cancer, it also abolishes the binding of HIV-1 to DC-SIGN by high-affinity binding fucose-rich glycans on semen clusterin to DC-SIGN [74].

DC-SIGN is well known for binding mannose-rich carbohydrates [61] that are present on the surface of filoviruses [100,101], particularly in the mucin domain of GP1, a subunit of the GP, on the Ebola virus. Since this domain is characterized by a high content of N- and O-linked glycosylation, it is, among other factors, responsible for the immune evasion of EBOV as well as impaired immune activation [102,103]. Some studies showed that DC-SIGN enhances the infection with the Ebola virus but is not directly involved in the entry of the virus [101,104]. Moreover, like in HIV, trans-infection is also possible through DC-SIGN [87]. Lasala et al. (2003) developed a glycodendritic structure called BH30sucMan which inhibits DC-SIGN-mediated EBOV infection by impeding their interaction [105]. In general, it was shown that glycodendrinanoparticles are able to mimic pathogens, thereby inhibiting Ebola infection of DCs by blocking viral binding to DC-SIGN [106].

Furthermore, DC-SIGN may play a role in the viral entry of SARS-CoV-2 [55] and as a potent trans-receptor [107] by binding high-mannose and Lewis epitopes on the spike protein. Recently, it was demonstrated that the same glycomimetic antagonist of DC-SIGN used against HIV, Polyman26, inhibits the interaction of the spike protein with the lectin receptor and thus hinders DC-SIGN-mediated SARS-CoV-2 trans-infections [107].

## 4. Selectins

Selectins belong to group IV of the CLRs and can be divided into three subgroups (E-, L-, and P-selectins) depending on their expression pattern (endothelial cells, leukocytes, or platelets) [108,109]. They consist of a N-terminal C-type lectin domain, an epidermal growth factor (EGF)-like domain, several regulatory short consensus repeats (SCRs), as well as a transmembrane domain and a cytoplasmic tail [110]. All three receptors recognize a minimal recognition pattern called sialyl Lewis antigen x (sLe^x^) as well as its isomer, sialyl Lewis antigen a (sLe^a^), comprising sialic acid and fucose (Siaα2,3Galβ1,4(Fucα1,4/3) GlcNAc) [111,112] (Figure 2c). SLe^x^ is presented by several GPs including the P-selectin glycoprotein ligand-1 (PSGL-1), which is a key ligand for all selectins [113].

The main difference between the three selectins lies in their expression patterns: E-selectin is expressed on stimulated endothelial cells, whereby the expression is induced upon inflammatory cytokines and can take several hours [114]. P-selectin, on the other hand, is constitutively expressed in platelets and endothelial cells and is rapidly translocated to the cell surface during inflammation [114]. Meanwhile, L-selectin is constitutively expressed on leukocytes [110] but is cleaved by the metalloproteinase ADAM17 upon cell activation in a process called L-selectin shedding [115,116].

All three receptors are suggested to be essential for inflammation [117]. By binding to glycan structures on leukocytes, the endothelial P- and E-selectins promote an initial tethering and rolling of leukocytes resulting in leukocyte extravasation [118,119,120]. L-selectin, on the other hand, might enhance inflammation by promoting leukocyte–endothelial [121] as well as leukocyte–leukocyte interactions [122]. Additionally, it was suggested that this binding process triggers signal transduction resulting in leukocyte integrin activation (P-selectin [123] and E-selectin [124]), cytokine secretion (P-selectin [125]), or neutrophil activation (L-selectin [126]). Moreover, it was indicated that P-selectin on platelets is involved in thrombus formation [127] and is essential for hemostasis [128,129]. L-selectin on lymphocytes promotes extravasation to lymphoid tissues by attaching to high endothelial venule cells on secondary lymphoid organs [130].

### 4.1. Selectins in Cancer

As selectins are physiologically important for the interactions involving leukocytes, platelets, or endothelial cells [109], it is not surprising that they play an essential role in tumor-promoting inflammation and cancer metastasis [131]. In particular, E-selectin was reportedly discovered to be upregulated during metastasis [132], representing an essential receptor in leukemia [133], and myeloma but also in solid tumors including pancreatic, prostate, colon, and breast carcinoma (reviewed in [134]). Moreover, there is strong evidence that the expression of sLe^x^ and sLe^a^ is also increased in many cancer types, for example, in gastrointestinal cancer, carcinoma or melanoma cells correlating with enhanced metastasis and poor prognosis [112,135,136,137]. The interaction between sLe^x^ and E-selectin is well recognized to enhance the adhesion of cancer cells to the endothelium [138], one example being leukemic cells adhering to the bone marrow [139,140]. Additionally, E-selectin overexpression and the subsequent interaction with the tumor-microenvironment was shown to activate pro-survival and antiapoptotic signaling via the ERK/AKT, PI3K, NF-κB, or Wnt pathway (reviewed in [134]), which promoted tumor progression, chemoresistance [134], and also the maintenance of cancer cell stemness [141]. In particular, its role in acute myeloid leukemia (AML) is well researched. It was discovered that AML blasts secrete inflammatory mediators leading to E-selectin upregulation [133]. This creates a protective vascular niche and induces pro-survival signaling via the AKT/NF-κB/mTOR pathway, which promotes cancer cell survival and regeneration as well as resistance to chemotherapy [133].

While the importance of the E-selectin-sLe^x^ interaction for promoting adhesion in tumor metastasis is well established, it is proposed that P- and L-selectin are essential for creating an inflammatory metastatic microenvironment once cancer cells intravasate and circulate in the bloodstream (reviewed in [131]). It is suggested that directly after intravasation or an initial arrest, P-selectin mediates the interaction between platelets and cancer cells, creating a tumor embolus [142]. As a consequence of cytokine and chemokine secretion, L-selectin recruits leukocytes to activated endothelial cells, which strengthens the embolus. This embolus further supports metastasis by enhancing the microvascular arrest, protecting tumor cells from immune cleavage, and promoting angiogenesis and cancer growth [131]. Moreover, recruited leukocytes can help tumor cells break the endothelial barrier and escape the bloodstream to invade other organs and tissues [143,144]. 

Currently, a glycomimetic of the sLe antigens named Uproleselan, or GMI-1271, seems very promising in selectively blocking the interaction between leukemic cells and E-selectin [145]. By impeding the adhesion of cancer cells to bone marrow, Uproleselan mobilizes cancer cells to the blood circulation and thus sensitizes them for chemotherapy [133,139]. Moreover, Uproleselan has been suggested to also inhibit pro-survival signaling and cancer cell regeneration as well as to decrease therapeutic side effects due to specific targeting (reviewed in [134]). Currently, two phase III clinical trials are ongoing to evaluate the efficacy of Uproleselan in combination with different chemotherapeutic agents in newly diagnosed (NCT03701308) and relapsed/refractory (NCT03616470) adult AML patients [146]. Furthermore, Uproleselan proved to be preclinically efficient in leukemia, myeloma, pancreatic, colon, as well as breast cancer cells [134], and is currently being tested in a phase II trial (NCT04682405) in multiple myeloma (MM) patients. Moreover, molecular biosimilars have already been developed targeting, for example, CXCR4 in addition to E-selectin (GMI-1359) [147].

### 4.2. Selectins in Viral Infections

In HIV, the glycoprotein gp120 was shown to play an essential role in binding the L-selectin receptor on T cells. A controversial role of L-selectin was observed in promoting HIV entry on the one hand and reducing infection on the other hand [148]. Firstly, it was discovered that the interaction with L-selectin on CD4+ T cells mediates the adhesion and subsequent entry of HIV into the host cell [148]. It could be observed that the accessory protein of HIV called Vpr was able to increase the mRNA expression in primary CD4+ T cells [149]. However, it was also shown that the shedding of L-selectin after the viral entry was essential for HIV release. Additionally, L-selectin shedding could avoid the transport of the T cells back to the lymphoid organs [115,148]. In this context, the HIV proteins Nef and Vpu were implied in sequestering L-selectin to reduce the cellular presentation of the receptor [150]. Inhibiting the shedding of L-selectin by inhibiting the matrix metalloproteinase ADAM17 with Batimastat was able to inhibit the release of viral particles without affecting viral entry. Therefore, blocking L-selectin shedding could potentially inhibit further HIV proliferation [148]. 

The protective character of L-selectin was also observed in influenza virus and vaccinia infections, where L-selectin was required to bring CD8+ T cells to the infection site and eliminate the virus [151]. After L-selectin is shed upon T cell priming, it is possibly re-expressed when CD8+ T cells migrate to lymph nodes in order to reach virally infected organs. Genetically engineering CD8+ T cells to express non-cleavable L-selectins could promote viral clearance [115,152]. Thus, CAR-T cells containing a modified L-selectin receptor could represent a possible therapy. In comparison to L-selectin, the importance of E-selectin in viruses, especially HIV, is much more debated [153]. In HIV, it was shown that the Tat protein was secreted by infected cells and, once taken up by healthy cells, could lead to the upregulation of E-selectin in these cells. This upregulation was suggested to enable HIV transfection of the brain [154]. However, E-selectin does not correlate with disease progression, and Hoffman et al. (2018) proposed an increase in E-selectin in an acute inflammatory, rather than chronic, phase of HIV infection [153].

Lastly, the ligands sLe^x^ and PSGL-1 might also be essential and could present future therapies or therapy targets. It was shown that sLe^x^ was enhanced in infected and transcriptionally active cells, enabling higher trafficking of the cells [155]. Meanwhile, additional PSGL-1 on virus-producing cells could compete with HIV but also SARS-CoV-2 for the selectin receptors. They sterically blocked the interaction and, thus, impeded virus attachment and infectivity [156,157].

## 5. Galectins

Galectins, first known as S-lectins, are a family of animal lectins the binding of which is mediated via a conserved CRD of approximately 130 amino acids [158,159]. Among the 15 galectins identified in mammals so far, 12 are also present in humans [160,161]. Depending on their presentation of CRD domains, galectins can be divided into three groups [162]. ‘Prototype’ galectins possess one CRD and can occur as monomers (gal-5, 7, 10) or dimers (gal-1, 2, 11, 13, 14). In addition, there are the ‘tandem-repeat type’ (gal-4, 6, 8, 9, 12), galectins with two CRDs linked by a short linker region. The third ‘chimeric type’ group, which only includes gal-3, contains one CRD and one non-CRD domain [162] (Figure 2d). In this review, we mainly concentrate on gal-1,3, and 9, as these galectins are considered to play a key role in cancer as well as in the context of viral infections [163,164].

Galectins exhibit affinity for β-galactose-containing glycoconjugates [158] with the minimal binding motif of *N*-acetyllactosamine (LacNAc, Gal-β (1,4)-GlcNAc) [165,166]. Therefore, LacNAc can occur as the terminal disaccharide of N-glycan chains or as a repeating unit in a polyglycan chain on N- or O-glycans [159,167,168,169]. However, the individual galectins show specificity towards certain variants of this minimum disaccharide ligand. While gal-3 preferentially binds to internal and terminal LacNAc residues of oligosaccharides, the binding of gal-1 is limited to terminal LacNAc residues [170]. In contrast, the two CRDs of gal-9 display affinity for repeated oligolactosamines as well as for branched N-glycans [171]. The ability to homodimerize and oligomerize enables galectins to simultaneously interact with several glycoconjugates due to bi- or multivalency [172,173]. In mammals, galectins are expressed by various immune cells, such as DCs [174], macrophages [175], mast cells [176], eosinophils [177,178], neutrophils [179], NK cells [180], B, and T cells [181,182,183,184]. It is possible that individual galectins have a wide range of effects on different cell types, but also that multiple galectins bind to and act on the same cell [185].

Galectins have a multitude of functions both intracellularly [186] and extracellularly [187,188,189,190]. Therefore, galectins are responsible for the regulation of important cellular processes like adhesion [191,192], differentiation [185], maturation [185], activation [192], proliferation [191,192], migration [192], trafficking [191], apoptosis [191,192], cytokine secretion [185,191], and cell–cell communication [185,191]. As a result, human galectins play multiple roles in a wide range of physiological functions, and are often directly linked to immunity and pathological processes such as cancer and viral infection [161,164,193].

### 5.1. Galectins in Cancer

Within the galectin family, gal-1, gal-3, gal-7, gal-8, gal-9, gal-10, and gal-12 have been linked to cancer so far [194]. In the following sections, the focus will be on gal-1, gal-3, and gal-9, as these galectins are among the most involved galectins in cancer hallmarks, according to current research [163].

#### 5.1.1. Galectin-1 and Galectin-3

Altered galectin expression is frequently present in cancer, with gal-1 being overexpressed in most cancer types, whereas expression differs more strongly in gal-3 (reviewed in [195]). In addition, tumor cells display an increase in the size and branching of N-glycan structures [13], leading to more terminal LacNAc residues and poly-LacNAc chains. This results in additional ligands for galectins, causing an enhanced affinity for most galectins, including gal-1, gal-3, and gal-9 [196,197,198].

According to Girotti et al. (2020), gal-1 and gal-3 promote proliferation, immune escape, replicative immortality, cell death resistance, angiogenesis, invasion, and metastasis. In addition, these galectins have both a promoting and an inhibitory effect on tumor suppressors such as TP53 [199,200,201]. Moreover, studies showed that gal-1 exhibits both effects in tumor-promoting inflammation [202,203] and gal-3 in genomic instability and mutation [204,205]. Furthermore, gal-3 supports tumor-promoting inflammation and impedes the deregulation of cellular energetics [206,207].

Blocking gal-1 and gal-3 with peptide- and carbohydrate-derived or small-molecule-specific inhibitors revealed a restraining effect on tumor growth, angiogenesis, and metastasis in preclinical as well as clinical studies (reviewed in [194]). Inhibition of gal-3 was found to disrupt multidrug resistance mechanisms leading to increased sensitivity to chemotherapeutic agents [208,209]. So far, different galectin inhibitors have been investigated in combination with chemotherapy, radiotherapy, or other immune interventions in various clinical trials [194,210,211,212]. In a phase II clinical trial (NCT00110721), the galectin inhibitor Davanat with high selectivity for gal-1 but only moderate selectivity towards gal-3 [213,214] showed an increase in life expectancy as well as a reduction in serious side effects in colorectal cancer patients [215]. Phase III of this clinical trial seems to have been accepted by the FDA but has not yet started [210]. Moreover, other phase II clinical studies (NCT00514696, NCT01681823) could confirm an increase in therapeutic effect [194,210]. In addition, there is an ongoing phase I trial (NCT02575404) of a galectin inhibitor in combination with a T cell checkpoint inhibitor. Overall, the large number of trials in this area indicate that targeting these galectins is still highly relevant as a potential cancer therapy.

#### 5.1.2. Galectin-9

In contrast to gal-1 and gal-3, the expression of gal-9 in cancer tissues is still understudied. Up to now, gal-9 expression has been found to be downregulated in most tumors [216,217,218,219,220], excluding oral cancer [221], pancreatic cancer [222], and Hodgkin’s lymphoma [223]. Intracellular and extracellular gal-9 has multiple functions. While intracellular gal-9 was found to be involved in cell adhesion, migration, proliferation, apoptosis, and trafficking, extracellular gal-9 was recently identified to be a multi-faceted immunomodulator [224]. On the one hand, extracellular gal-9 downregulates the response of effector Th1 cells and induces their cell death by specifically binding to T cell immunoglobulin and mucin domain-containing molecule 3 (TIM-3), a membrane protein on differentiated Th1 cells [225,226,227,228,229]. On the other hand, extracellular gal-9 showed an involvement in DC maturation and increased the differentiation and suppressive activity of regulatory T cells [230].

This two-sided role of gal-9 is also reflected in tumors [211]. So far, it has been shown that gal-9 promotes immune escape, angiogenesis, and tumor growth, while on the other hand, gal-9 also inhibits tumor energy metabolism [163]. Existing literature indicates that, depending on which ligand gal-9 interacts with on T cells, the APCs or tumor cells it exhibits either an antitumor effect or promotes tumor activity [211]. The binding of gal-9 to N-glycans on TIM-3 in myeloid leukemia cells restricts tumor growth and metastasis [231]. Similarly, immunohistochemical studies of samples from patients with hepatocellular carcinoma, breast cancer, melanoma, and cervical squamous-cell carcinoma suggest that gal-9 suppresses metastasis and, thus, may be used as a marker for the metastatic potential of these tumors [217,219,220,232]. In contrast, the results of mouse models (in vivo/in vitro) suggested an apoptotic effect of gal-9 on tumor cells in chronic myeloid leukemia (CML), malignant melanomas, and different gastrointestinal and urogenital tumors [233,234,235,236,237,238,239,240,241,242]. Furthermore, Zhang et al. (2020) identified that intracellular as well as extracellular gal-9 plays an important role in promoting immunosuppressive myeloid cells in tumors. It was confirmed that high levels of gal-9 in the tumor tissue or serum of cancer patients are associated with an unfavorable outcome [224]. Recently, a new molecular pathway has been uncovered in which gal-9 plays a key role in combination with PD-1, making it a target for cancer immunotherapy [243]. Gal-9 was found to interact with PD-1 to inhibit the apoptosis of TIM-3 T cells and further promote the persistence of exhausted T cells in tumors. Furthermore, gal-9 expression was shown to be upregulated by interferon-β and secretion by a combination of interferon-β/γ [243]. Moreover, it was demonstrated in vitro that the inhibition of gal-9-TIM-3 interaction using the anti-gal-9 antibody RG9-1 leads to the suppression of gal-9-induced T cell death. However, only the combination of this gal-9 antibody with an agonistic antibody against the co-stimulatory receptor GITR (glucocorticoid-induced tumor necrosis factor receptor-related protein) exhibited an antitumor effect and prolonged survival [243]. There was also prior evidence that the inhibition of the TIM-3-gal-9 pathway leads to increased T cell proliferation and the secretion of cytokines [227]. Therefore, LYT-200, a monoclonal antibody targeting gal-9, is currently being tested in phase I/II of a clinical trial (NCT04666688) in combination with chemotherapy or anti-PD-1 in patients with metastatic solid tumors. Taken together, previous insights in this field suggest that targeting gal-9 is an effective and promising strategy for cancer immunotherapy.

### 5.2. Galectins in Viral Infections

While so far gal-1, 3, 8, and 9 were reported to play a key role in virus attachment, entry, replication, and immune response (reviewed in [164]), we focus here only on gal-1, gal-3, and gal-9 in HIV and influenza virus infections. 

#### 5.2.1. Galectin-1

The role of gal-1 in HIV infection is reflected by its expression in the thymus [244,245] and lymph nodes as well as its secretion by activated CD8+ T lymphocytes, which are present at high levels in HIV-1-positive patients [246]. One key function of gal-1 is the stabilization of HIV-1 attachment to host cells which, by shortening the time required to establish infection, may facilitate HIV-1 infectivity [246,247]. Moreover, the altered surface glycosylation of HIV-1 infected T cells, such as the augmented expression of LacNAc residues, leads to the enhanced susceptibility to gal-1-induced cell death of peripheral blood lymphocytes and may be linked to T cell loss in AIDS [248]. Inhibition of gal-1 with three lactoside derivatives was successful in blocking gal-1-mediated, increased HIV-1 attachment and infectivity [249].

Gal-1 exhibits a completely opposite mode of action in influenza virus infection. Gal-1 is assumed to inhibit influenza virus infection by binding to hemagglutinin and, possibly, neuraminidase, two important surface GPs of this virus that are essential for virus attachment and release, respectively [250]. A study in mice revealed that intranasal treatment with human-recombinant gal-1 could reduce viral load, inflammation, and apoptosis in the lungs [250]. Thus, the administration of gal-1 may represent a new strategy in the therapy of influenza viruses.

#### 5.2.2. Galectin-3

In the search for a link between HIV and gal-3, it was discovered that the Tat protein of HIV-1, which is crucial for viral replication, induces the upregulation of gal-3 in various human cell types [251,252]. Later, gal-3 was found to facilitate HIV-1 release by stabilizing proteins important for this process [253]. In contrast, gal-3 was also observed to promote the elimination of HIV-1-infected macrophages through caspase-independent cell death [254].

To date, not much is known about the effect of gal-3 on influenza virus infection. Anthraquinones such as aloe-emodin exhibit antiviral activity as well as an inhibitory effect on the replication of various viruses [255,256,257,258]. In the case of influenza virus infection, it was observed that treatment with aloe-emodin led to an upregulation of gal-3 [259]. This recombinant gal-3 in turn increased the expression of antiviral genes such as RNA-dependent protein kinase and interferon-β/γ [259].

SARS-CoV-2 belongs to the genus β-coronaviridae. It was found that the key domain of the spike protein in this genus is morphologically very close to human gal-3 [260,261]. Consequently, gal-3 inhibitors could potentially hamper virus entry [262]. Furthermore, gal-3 inhibition was demonstrated to suppress host inflammatory response [262,263,264]. On top of that, the gal-3 inhibitor TD-139, currently being tested in phase IIb clinical trials, showed promising results [265]. In this context, it should be noted that a gal-1 inhibitor against COVID-19 is in development, which is expected to perturb viral attachment but also to manipulate cytokine release [262].

#### 5.2.3. Galectin-9

Gal-9 promotes HIV-1 transcription and replication through alternative glycosylation [266] and T cell receptor-dependent ERK signaling as well as CREB pathways [267]. Infection with HIV-1 increases gal-9 levels, which may contribute to NK cell dysfunction in chronic HIV infection [268]. Furthermore, it was found that gal-9 regulates the host restriction factor p21 [269], which modulates HIV transcription in patients receiving antiretroviral therapy [270]. As a result, Abdel-Mohsen et al. (2016) showed that a recombinant form of gal-9 could reverse the latency of HIV in vitro. In addition, the glycosylation of infected host cells was found to mediate signals that could determine the infectivity of HIV [271].

In influenza virus infections, it could be observed that infected patients have a higher gal-9 expression in plasma, making gal-9 a potential novel biomarker in this field [272]. Moreover, it was revealed that the inhibition of gal-9 signaling leads to the activation of the virus-specific CD8 T cell response. This caused the increased production of virus-specific antibodies and supported rapid virus clearance [273]. 

## 6. Conclusions

In this review we have considered the glycan–lectin interactions for the four different lectin subtypes MGL, DC-SIGN, selectins, and galectins physiologically and both in cancer and in viral infections. We have summarized some approaches to disrupt them in order to see whether there are commonalities in the interactions between cancer and viral infections. The main findings for the four lectin subtypes are:

MGL binds galactose-terminated glycans and GalNAc, which mediates the internalization and presentation of pathogenic molecules during immune response. However, cancer cells and viruses have evolved strategies to avoid the initial role of MGL in immunity activation and even use MGL to their advantage. In cancer, MGL interacts with truncated O-glycans, namely the (s)Tn and (s)T antigen on glycan carriers like MUC1. This increases anti-inflammatory cytokine levels and impairs the differentiation of DCs and the Th cell-mediated response, causing the immune evasion of cancer cells. Currently, Tn-MUC-1 CAR-T cells and cancer vaccines targeting Tn-MUC1 are investigated preclinically. In viral infections, on the other hand, MGL was shown to interact with terminal galactose on the influenza virus or the spike protein of SARS-CoV-2, functioning as a potential entry receptor. Moreover, it interacts with the viral envelope protein of GP on EBOV enhanced infectivity, which could be reduced with the injection of GP-neutralizing antibodies.

DC-SIGN binds to high-mannose and fucose glycans like the Lewis-antigen family, which promotes the detection of pathogens and T cell presentation. In cancer cells, overexpressed Lewis antigens on glycan carriers like CEA interact with DC-SIGN. The effect is similar to that of MGL. Immunosuppressive cytokines prevent DC maturation, which makes them unable to promote a T cell response against cancer cells. Apart from immune evasion, the DC-SIGN-glycan interaction promotes cancer carcinogenesis and tumor progression. Several therapeutic methods target the DC-SIGN receptor using glycan-modified antigens and antibodies either against DC-SIGN or Le^y^. During viral infections, DC-SIGN was shown to bind gp120 on HIV using DC as a shuttle for the trans-infection of CD4+ T cells. Moreover, the GP1 of EBOV binding to DC-SIGN could support immune evasion, thus enhancing viral infection. Glycodendritic structures mimicking pathogens could block these viral interactions with DC-SIGN. Lastly, the glycomimetic antagonist of DC-SIGN, named Polyman26, was shown to inhibit both DC-SIGN-mediated SARS-CoV-2 and HIV infection.

All three selectin subtypes (E-selectin, L-selectin, and P-selectin) bind sLe^x/a^ carried, for example, by PSGL-1 promoting inflammation and hemostasis. In cancer, selectins binding to overexpressed sLe^x^ were shown to be essential for enabling cancer cell attachment during metastasis as well as for creating a metastatic tumor embolus promoting immune evasion. Currently, one of the most promising therapies is the sLe^x^-glycomimetic Uproleselan, which blocks E-selectin and is being tested in two phase III studies. Meanwhile, the importance of L-selectin in virus is a little more researched, especially in HIV. While the interaction with gp120 was shown to promote HIV entry, the shedding of the receptor was required for viral release. In influenza virus infection, L-selectin was required for the CD8+ T cells to eliminate the virus. Therefore, current approaches try to inhibit L-selectin shedding by using Batimastat or genetically engineered CD8+ T cells. Moreover, the addition of selectin ligands like PSGL-1 could compete with viruses like HIV or SARS-CoV-2 for the receptors and impede infectivity. Thus, it might also be interesting to test whether the sLe^x^-glycomimetic Uproleselan has an effect in viral infections.

Galectins interacting with LacNAc and β-galactose-glycoconjugates were shown to be essential in many cellular processes ranging from the maintenance of cell homeostasis to immune and inflammatory responses. In cancer, the increased branching of N-glycans and, partially, galectin overexpression enhances galectin affinity to the cancer cells promoting immune escape and tumor proliferation as well as a range of other cancer hallmarks. However, the interaction with galectins also exhibited antitumor effects depending on the specific glycan ligands. A series of clinical trials testing peptide-derived, carbohydrate-derived, or small molecule gal-1 and gal-3 inhibitors, some in combination with chemotherapy or radiotherapy, show promise to block the interaction. Moreover, recent approaches indicate that the inhibition of gal-9 combined with other therapeutic modalities is a promising strategy in cancer immunotherapy. In viral infections, galectins have a more controversial role. While gal-1 and gal-3 interactions with viral GPs were shown to facilitate attachment and release in HIV, they inhibited infection with the influenza virus. Thus, gal-1 was blocked in HIV and also SARS-CoV-2 to reduce the infection while, in the influenza virus infections, recombinant gal-1 was administered.

In summary, it has been established that the four lectin subtypes MGL, DC-SIGN, selectins, and galectins play an essential role in cancer and viral infections by interacting with specific glycans. However, research on the detailed mechanisms of these glycan–lectin interactions and potential therapies is still at an early stage. So far, the therapeutic approaches look very promising, but it remains to be seen whether some of them will be successful in the clinical phases. While comparing the interactions between lectins and glycans in cancer and viral infections and the ways to disrupt them, we have not yet found commonalities suggesting that promising therapeutic approaches in one disease can be translated to another. Further research in this direction must be conducted.

## Abbreviations

AML	acute myeloid leukemia
APC	antigen-presenting cell
CAR	chimeric antigen receptor
CEA	carcinoembryonic antigen
CLR	C-type lectin receptor
CML	chronic myeloid leukemia
CRD	carbohydrate recognition domain
CTL	cytotoxic T-lymphocyte
CTLD	C-type lectin-like domain
DC	dendritic cell
DC-SIGN	DC-specific intracellular adhesion molecule 3-grabbing nonintegrin
EBOV	Ebola virus
EGF	epidermal growth factor
GalNAc	α-*N*-acetylgalactosamine
GP	glycoprotein
HIV	human immunodeficiency virus
ICAM3	intracellular adhesion molecule 3
iDC	immature dendritic cell
IL	interleukin
LacNAc	*N*-acetyllactosamine
Le	Lewis antigen
MGL	macrophage galactose-binding lectin
MHC	major histocompatibility complex
MUC1	mucin 1
NK cell	natural killer cell
PSGL-1	P-selectin glycoprotein ligand-1
SARS-CoV-2	severe acute respiratory syndrome coronavirus 2
SCR	short consensus repeat
Th cell	T-helper cell
TIM-3	T cell immunoglobulin and mucin-domain containing 3
